# Atorvastatin treatment improves effects of implanted mesenchymal stem cells: meta-analysis of animal models with acute myocardial infarction

**DOI:** 10.1186/s12872-015-0162-6

**Published:** 2015-12-14

**Authors:** Guo Dai, Qing Xu, Rong Luo, Jianfang Gao, Hui Chen, Yun Deng, Yongqing Li, Yuequn Wang, Wuzhou Yuan, Xiushan Wu

**Affiliations:** The Center for Heart Development, Key Laboratory of MOE for Developmental Biology and Protein Chemistry, College of Life Sciences, Hunan Normal University, Changsha, Hunan 410081 P. R. China; The Center for Heart Development, Hunan Normal University, Changsha, 410081 Hunan P. R. China

**Keywords:** Meta-analysis, Acute myocardial infarction, Animal models, Cell therapy, Mesenchymal stem cells

## Abstract

**Background:**

Previous studies reported that Atorvastatin (ATOR) can improve the efficacy of Mesenchymal stem cells (MSCs) transplantation after acute myocardial infarction (AMI). However, the results of those studies were inconsistent. To clarify the beneficial effects of atorvastatin added to the cell therapy with MSCs in animal model of acute myocardial infarction (AMI), we performed a systematic review and meta-analysis of case–control studies.

**Methods:**

Searches were performed using the PubMed database, the Excerpta Medica Database (Embase), the Science Citation Index, the China National Knowledge Information database, the Wanfang database, and the Chinese Scientific and Technological Journal Database (VIP database). The search term included “Atorvastatin (or Ator)”, “Mesenchymal Stem Cells (or Mesenchymal Stem Cell or MSC or MSCs)” and “Acute Myocardial Infarction (or Myocardial Infarction or AMI or MI)”. The endpoints were the left ventricular ejection fraction (LVEF) in animal model with AMI.

**Results:**

In total, 5 studies were included in the meta-analysis. Pooled analysis indicated a significant LVEF difference at 4 weeks follow-up between MSCs + ATOR combine group and MSCs alone group (95 % CI, 9.09–13.62 %; *P* < 0.01) with heterogeneity (*P* = 0.28; *P* >0.05) and inconsistency (I^2^: 22 %).

**Conclusions:**

Atorvastatin can enhance the existing effects of MSCs transplantation, and this combinational therapy is a superior cell/pharmacological therapeutic approach that merits future preclinical and clinical studies.

## Background

Acute myocardial infarction (AMI) is the leading cause of death among people in industrialized nations [1]. Although early revascularization can save part of ischemic myocardium, necrotic myocardial cells, which cannot regenerate, will gradually be replaced by scar tissue, leading to ventricular remodeling and heart failure, and thus seriously affect the survival rate and quality of life of survivors [2]. Despite the rapid development of therapeutic techniques and ideas, the treatments of heart failure secondary to AMI are still very limited, among which stem cell transplantation is one of the most promising [3].

Due to no strong differentiation of immune rejection and easy to get, the bone marrow-derived mesenchymal stem cells (MSCs) is one of the best sources of transplanted cells. Therefore, MSCs have been widely utilized as a result of their plasticity, availability, and lack of immunological rejection or ethical issues [4]. However, many studies have demonstrated the poor survival and retention of transplanted cells in vivo, whether this is due to properties of the cells themselves, the extremely hostile microenvironment in the per infarct region, or a combination of both [5]. For these reasons the focus has been on efforts to improve the tolerance of stem cells to the adverse microenvironment, which would hopefully lead to the development of a clinical approach to improve stem cell survival and tissue repair capacity [6].

Recent studies have demonstrated that combined therapy with MSCs and atorvastatin (ATOR), a blood cholesterol-lowering agent, produces synergistic beneficial effects in the treatment of AMI [7]. However, the number of experimental animals in most of studies selected is limited. In addition, many large animal studies in AMI and ischaemic cardiomyopathy have been conflicting outcomes. We hypothesize that meta-analysis of these experimental data might be helpful to design future clinical studies similarly to the meta-analysis of human cardiac stem cell trials.

We performed a systematic overview of the pertinent literature including a quantitative meta-analysis to assess the effects of stem cell transplantation in animals with acute myocardial infarction. Combined MSCs therapy and pharmacotherapy is one of these proof-in-principle approaches.

## Methods

### Search strategy

The following databases were searched in Dec 2014: PubMed database, the Excerpta Medica Database (Embase), the Science Citation Index, the China National Knowledge Information database, the Wanfang database, and the Chinese Scientific and Technological Journal Database (VIP database).

For the association of ATOR, Mesenchymal Stem Cells and Acute Myocardial Infarction, the following search term were used in searching the previous database: “Atorvastatin (or Ator)”, “Mesenchymal Stem Cells (or Mesenchymal Stem Cell or MSC or MSCs)” and “Acute Myocardial Infarction (or Myocardial Infarction or AMI or MI)”. No language is limited. In addition, the references of retrieved articles were also screened to find the related papers. In addition, we performed manual searches by scanning the reference lists of the selected articles to locate additional papers related to the topic.

### Study selection

Two investigators independently reviewed all studies and extracted the data using a standard information extraction and reached consensus on all items. Only those articles that investigated the effect of ATOR combined with mesenchymal stem cell transplantation on cardiac function in animals with acute myocardial infarction were included. Reviews, editorials, comments, reports from scientific sessions and discussions were excluded. We obtained the full text of articles that were identified as either relevant or possibly relevant, based on their titles and abstracts.

### Quality assessment and data extraction

The quality of studies was independently assessed by two reviewers using a risk of bias assessment by van der Spoel TI’s studies [8]: including randomization (yes/no), adequate allocation (y/n), adequate method of randomization (y/n), blinding of the operator (y/n), and blinding of the functional analysis (y/n). The following information was extracted from the complete manuscripts of the qualified studies: basal characteristics of the study, the left ventricular ejection fraction (LVEF).

### Statistical analysis

Our primary outcome was difference in mean LVEF (reported in %) at follow-up between mesenchymal stem cells transplantation group (MSCs group) and mesenchymal stem cells treated with ATOR transplantation group (MSCs + ATOR group). In case of multiple measurements over time, data measured at the longest duration of follow-up were used for analysis. A random-effect model was applied. Continuous variables were reported as weighted mean differences (WMD) with 95 % confidence intervals (CI) between the cell-treated animals and control groups. In case of data, the pooled estimate of effect was presented as odds ratio (OR) with 95 % CI [9]. Inconsistency was estimated by using the I^2^ statistic; values of 25, 50, and 75 % were considered low, moderate, and high inconsistency, respectively [10]. Sensitivity analysis was also performed to test the robustness of the results by excluding a study one by one. All analyses were performed with Review Manager version 5 (The Nordic Cochrane Center, København, Denmark) and IBM SPSS Statistics 19 (SPSS, Chicago, IL, USA).

## Results

### Search results

**Fig. 1:**
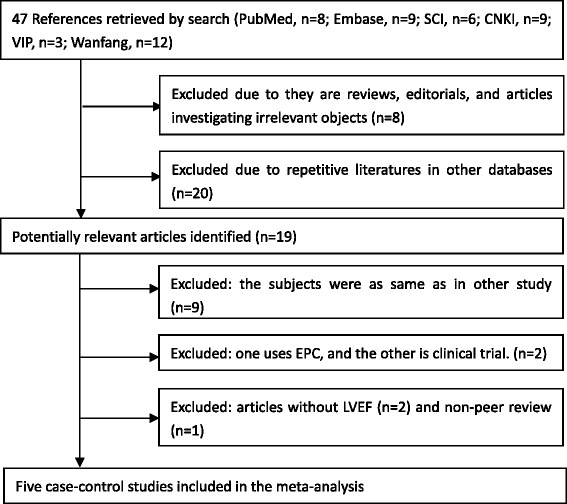
Flowchart of enrolled studies on cell therapy in animals with acute MI

Totally forty-seven references were retrieved. Among them, twenty were repetitive literatures in other databases; eight literatures were excluded because they are reviews, editorials, and or comments. In the end, five case–control studies were included in the meta-analysis. Figure 1 showed the flow diagram of studies selection.

### The quality of studies

The five studies all established an AMI animal model by performing thoracotomy and ligating the left descending coronary artery, and then randomly divided them into three groups: one group of AMI control group, one group of MSC transplantation group, and the third group conducted a joint ATOR and MSC transplantation group. Table 1 lists the eligible studies which included in as well as their main characteristics. Finally, within the four weeks after transplantation, five studies examined the left ventricular ejection fraction (LVEF) by echocardiography. Table 2 show the methodological quality of the enrolled studies. All studies reported the method of randomization, but did not indicate whether blinded analysis of LVEF. Table 3 shows the comparisons of cardiac function measured by echocardiography and hemodynamic examination in animal model of acute myocardial infarction of the enrolled studies.

**Table 1:** Characteristics of studies included in the Meta-Analysis

Author (year)	Language	Type of animal	Number of cells	Atorvastatin treatment	Route of delivery	Timing of cell therapy after MI
Zhang Q et al. (2014) [16]	English	Rat	5 × 10^6^cells/animal	10 mg/kg/day	Intramuscularly injection	4 weeks
Qu Z et al. (2013) [17]	English	Rabbit	4 × 10^7^cells/50uL	1.5 mg/kg/day	Intramuscularly injection	4 weeks
Song L et al. (2013) [18]	English	Swine	3 × 10^7^cells/animal	0.25 mg/kg/day	Intramuscularly injection	4 weeks
Cai A et al. (2011) [19]	English	Rat	1 × 10^6^cells /100uL	10 mg/kg/day	Intramuscularly injection	4 weeks
Yang YJ et al. (2008) [20]	English	Swine	3 × 10^7^cells/animal	0.25 mg/kg/day	Intramuscularly injection	4 weeks

**Table 2:** Methodological quality of the included studies

Study	RCT	Adequate allocation	Method of randomization described	Operator blinded	Analyst blinded
Zhang Q et al. (2014) [16]	Y	N	N	N	N
Qu Z et al. (2013) [17]	Y	N	N	N	N
Song L et al. (2013) [18]	Y	N	N	N	N
Cai A et al. (2011) [19]	Y	N	N	N	N
Yang YJ et al. (2008) [20]	Y	N	N	N	N

*RCT* Randomized trial, *Y* Yes, *N* No

**Table 3:** Comparisons of cardiac function measured by echocardiography and hemodynamic examination in animal model of acute myocardial infarction

Study	Type of animal	Control LVEF (%)	Number	MSCs LVEF (%)	n	Ator + MSCs LVEF (%)	Number
Zhang Q et al. (2014) [16]	Rat	48.1 ± 5.2	10	51.9 ± 2.4	10	65.3 ± 5.3	10
Qu Z et al. (2013) [17]	Rabbit	48.67	8	59.14	9	67.32	9
Song L et al. (2013) [18]	Swine	43.16 ± 8.02	6	48.75 ± 12.64	6	49.76 ± 12.09	6
Cai A et al. (2011) [19]	Rat	44.63 ± 3.22	8	46.17 ± 2.03	7	56.78 ± 3.66	7
Yang YJ et al. (2008) [20]	Swine	42.0 ± 7.1	6	41.3 ± 8.8	6	49.7 ± 10.4	7

*LVEF* (%) The left ventricular ejection fraction, (mean ± SD)

### Meta-analysis

**Fig. 2:**
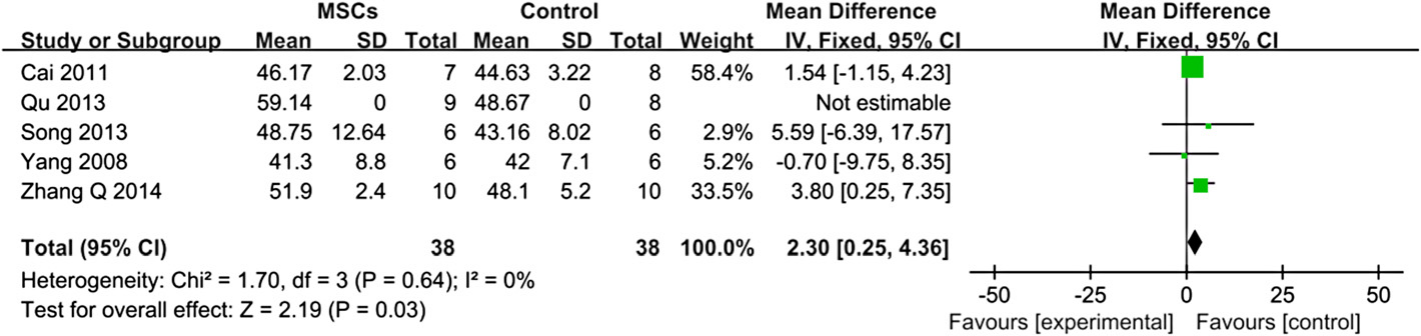
Forest plot showing the impact of MSCs therapy on LVEF improvement compared with controls

Within the four weeks after transplantation, five studies examined LVEF, including thirty-eight cases in AMI control group, thirty-eight cases of MSC transplantation group, and thirty-nine cases which conducted a joint ATOR and MSC transplantation. Firstly, comparing MSCs group and control group, it has been found a LVEF difference of 2.30 % at follow-up after MSCs group vs control (95 % CI, 0.25–4.36 %; *P* > 0.01) with inconsistency (I^2^: 0 %; Fig. 2), implying that there is no significant difference between MSCs group and control group.

**Fig. 3:**
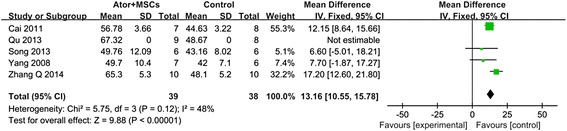
Forest plot showing the impact of MSCs + Ator therapy on LVEF improvement compared with controls

**Fig. 4:**
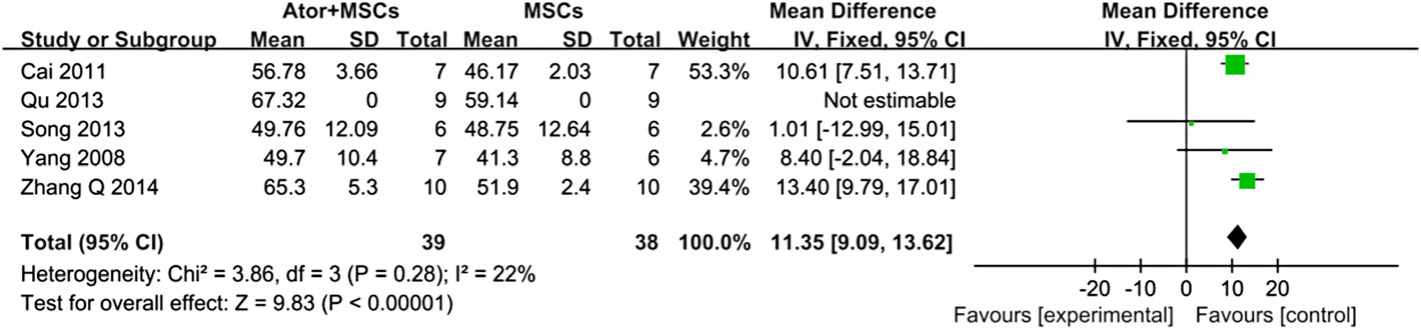
Forest plot showing the impact of MSCs + Ator therapy on LVEF improvement compared with that of MSCs therapy

As shown in Fig. 3, pooled analysis showed a LVEF difference of 13.16 % at follow-up after MSCs + ATOR group vs. control (95 % CI, 10.55–15.78 %; *P* < 0.01) with heterogeneity (*P* = 0.12; *P* > 0.05) and inconsistency (I^2^: 48 %). The results suggested that, compared with control, MSCs + ATOR contributes more to restoring myocardial infarction cardiac function. As shown in Fig. 4, pooled analysis showed a LVEF difference of 11.35 % at follow-up after MSCs + ATOR group vs. MSCs group (95 % CI, 9.09–13.62 %; *P* < 0.01) with heterogeneity (*P* = 0.28; *P* > 0.05) and inconsistency (I^2^: 22 %). The results suggested that, compared with MSCs transplantation alone, MSCs + ATOR contributes more to restoring myocardial infarction cardiac function.

**Fig. 5:**
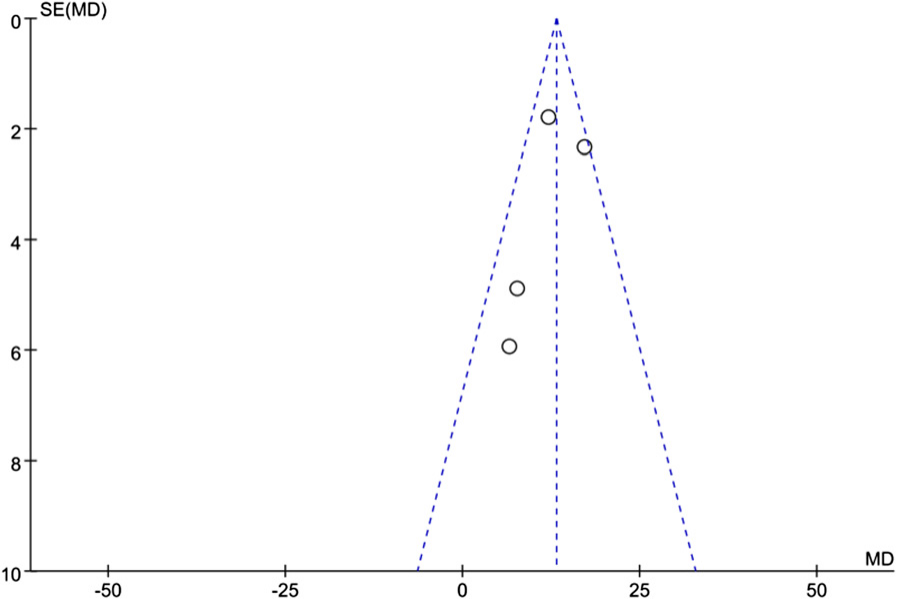
Funnel plot for LVEF improvement between MSCs + ATOR group and MSCs group. No evidence for publication bias was found. SE, standard error; MD, mean difference

In addition, Sensitivity analysis demonstrated that the result is same as before, indicated that the pooled meta-analysis results is very robust. The funnel plot for LVEF suggests a lack of publication bias as values were evenly distributed around the overall estimate (Fig. 5).

## Discussion

Although stem cells are studied clinically for cardiac repair, its effects are still controversial [11]. Some studies have shown that most stem cells were lost within 24 h of transplantation, only 15 % survived for 12 weeks. The quick loss after transplantation is mainly due to cell leak age out of the myocardium or wash-out through the vascular system [9]. Therefore, protection of graft cells from acute death in ischemic myocardium is important for clinical applications. Common statins include pravastatin, lovastatin, simvastatin and atorvastatin, which were used agents in patients with coronary heart disease owing to their superior ability to reduce blood cholesterols [12]. Previous studies showed that different types of statins play different roles in the induction of apoptosis of MSC. Lovastatin and atorvastatin have protective effect [13], while simvastatin can promote apoptosis [14]. The properties of Ator are well predicted to offer improvement of the microenvironment for implanted stem cells [15]. Among them most articles have explored the combination of atorvastatin and MSC to treat myocardial infarction.

The current analysis comprises data of five published studies involving animals with AMI, which treated with mesenchymal stem cells or Atorstatin + MSCs [16–20]. We first analyzed the therapy of AMI by MSCs transplantation alone. However, it was found compared with the control group, the recovery of ventricular function is limited after transplantation, which might be related to insufficient MSC’s survival rate. Subsequently, we analyzed the transplantation of ATOR combined with MSC, it showed compared with both the control group and MSC group, the ventricular function was significantly improved as reflected by the magnified restoration of the enlarged LVEF in AMI. The study showed the therapeutic effect of the transplantation of ATOR+ MSC is better than sole MSCs transplantation, which contributes to further clinical application of MSCs. Our data have demonstrated that atorvastatin enhanced MSCs-induced improvement of ischemic cardiac dysfunction, as reflected by the magnified restoration of the enlarged LVEF in AMI. This is to say, Ator can exert protective effects on the myocardium undergoing infarction and reperfusion injury in conjunction with MSC transplantation.

Limitations of this paper are in the following aspects: 1) the sample size is still relatively small, only including approximately 80 animals of different species. It is hoped that more researches can be incorporated; 2) the study failed to analyze the appropriate dose of the drug. During transplantation, different studies chose a different dose, but how much dose is the optimal needs further study. 3) The study didn’t cover the mechanism of Ator. This study researched the impact of Ator to the therapy of AMI, but failed to provide data analysis of the causes of the impact, for example, whether it is anti-apoptotic, pro-differentiation, etc., which needs further study. 4) Other statins may also have a similar effect, which is yet to be explored. 5) D’Ascenzo F’s study shows that remote ischaemic preconditioning (RIPC) can reduce the incidence of periprocedural myocardial infarction (PMI) following percutaneous coronary intervention (PCI), especially when performed in the lower limb and for patients with multivessel disease and complex lesions [21]. During the “ATOR + MSC” transplantation process, RIPC may have a synergistic effect, which needs to be further studied.

To the best of our knowledge, this is the first systematic review and meta-analysis in large animal models to evaluate the effect of cell therapy in ischaemic heart disease. This analysis showed that large animal models are valid to predict outcome of clinical trials. More-over, the results showed that cardiac cell therapy is safe, led to an improved LVEF, and revealed important clues for designing (pre-) clinical trials.

The reported benefits of stem cell therapy for cardiac function in clinical trials have been only modest. One of the unresolved issues is the rather rapid disappearance of cells after a few days, which is accompanied by the lack of any demonstrable regenerative effect.

## Conclusions

Atorvastatin can enhance the existing effects of MSCs transplantation, and this combinational therapy is a superior cell/pharmacological therapeutic approach that merits future preclinical and clinical studies.

## Abbreviations

AMI: Acute myocardial infarction
ATOR: Atorvastatin
Embase: The Excerpta Medica Database
LVEF: The left ventricular ejection fraction
MSCs: Mesenchymal stem cells
VIP database: The Chinese Scientific and Technological Journal Database
